# Health Inequalities in Children and Adolescents: A Scoping Review of the Mediating and Moderating Effects of Family Characteristics

**DOI:** 10.3390/ijerph18157739

**Published:** 2021-07-21

**Authors:** Miriam Blume, Petra Rattay, Stephanie Hoffmann, Jacob Spallek, Lydia Sander, Raphael Herr, Matthias Richter, Irene Moor, Nico Dragano, Claudia Pischke, Iryna Iashchenko, Claudia Hövener, Benjamin Wachtler

**Affiliations:** 1 Department of Epidemiology and Health Monitoring, Robert Koch-Institute, 13353 Berlin, Germany; blumemi@rki.de (M.B.); rattayp@rki.de (P.R.); hoevenerc@rki.de (C.H.); 2 Department of Public Health, Brandenburg University of Technology Cottbus-Senftenberg, 01968 Senftenberg, Germany; stephanie.hoffmann@b-tu.de (S.H.); jacob.spallek@b-tu.de (J.S.); lydia.sander@b-tu.de (L.S.); 3 Mannheim Institute of Public Health, Social and Preventive Medicine, Medical Faculty Mannheim, Heidelberg University, 68167 Mannheim, Germany; Raphael.Herr@medma.uni-heidelberg.de; 4 Institute of Medical Sociology, Medical Faculty, Martin-Luther-University Halle-Wittenberg, 06112 Halle, Germany; m.richter@medizin.uni-halle.de (M.R.); irene.moor@medizin.uni-halle.de (I.M.); 5 Institute of Medical Sociology, Centre for Health and Society, Medical Faculty, Heinrich Heine University Düsseldorf, 40225 Düsseldorf, Germany; Dragano@med.uni-duesseldorf.de (N.D.); claudia.pischke@hhu.de (C.P.); 6 Health Economics, Technical University of Munich, 80992 München, Germany; iryna.iashchenko@tum.de

**Keywords:** health inequalities, socioeconomic position, health, health behavior, moderation, mediation, school-age parenting, parent-child relationship, family conflict

## Abstract

This scoping review systematically mapped evidence of the mediating and moderating effects of family characteristics on health inequalities in school-aged children and adolescents (6–18 years) in countries with developed economies in Europe and North America. We conducted a systematic scoping review following the PRISMA extension for Scoping Reviews recommendations. We searched the PubMed, PsycINFO and Scopus databases. Two reviewers independently screened titles, abstracts and full texts. Evidence was synthesized narratively. Of the 12,403 records initially identified, 50 articles were included in the synthesis. The included studies were conducted in the United States (*n* = 27), Europe (*n* = 18), Canada (*n* = 3), or in multiple countries combined (*n* = 2). We found that mental health was the most frequently assessed health outcome. The included studies reported that different family characteristics mediated or moderated health inequalities. Parental mental health, parenting practices, and parent-child-relationships were most frequently examined, and were found to be important mediating or moderating factors. In addition, family conflict and distress were relevant family characteristics. Future research should integrate additional health outcomes besides mental health, and attempt to integrate the complexity of families. The family characteristics identified in this review represent potential starting points for reducing health inequalities in childhood and adolescence.

## 1. Introduction

Health and ill-health are unequally distributed between and across different populations, with individuals in more socially disadvantaged positions typically experiencing worse health outcomes than their better-off counterparts [1]. Over the last several decades, social-epidemiological research has revealed a correlation between socioeconomic position (SEP) and various health outcomes at different life stages from early childhood [2] to older age [3]. Such health inequalities have consistently been found within and between different countries and at different points in time [4,5,6].

Health inequalities are most frequently described for non-communicable diseases that are generally more common among older people. However, many of these diseases also exist in younger people. Such inequalities have consistently been found for several indicators of ill-health during childhood and adolescence, such as being overweight, obesity, accidents, injuries and poor subjective general health, as well as psychosomatic and mental health problems [4,5,7].

In addition to these direct negative and inequitable impacts on children’s health and wellbeing, health inequalities among children and adolescents are of particular concern because various studies have shown that health developments during childhood have a substantial influence on health trajectories and health status in later life [8,9,10]. Thus, inequalities in health during childhood and adolescence might provide the foundation for health inequalities across the life course that eventually lead to unequal and potentially preventable loss of years of life. At the same time, ill-health during childhood is related to more severe social disadvantage in later life, such as fewer years of education and less advantageous employment conditions that could eventually lead to poorer health during the whole life course [9,10]. Health in children and adolescents has therefore become a major current focus of health promotion interventions.

Research on health trajectories across the life-course has emphasized the particular importance of prenatal [11] and early-childhood experiences for the development of health and disease [12], and this life stage has also been identified as a major target for tackling health inequalities [10]. Comparatively less research has focused on age-specific experiences of school-aged children and adolescents that might shape and sustain patterns of health and health inequalities. Therefore, we aimed to cover the existing research gap and focus our synthesis on school-aged children and adolescents (6–18 years).

Patterns of health inequalities are produced through a complex interplay between biology and the ecological as well as societal context of our lived experience [13]. Ecosocial [14] or social-ecological theories [15,16] are increasingly recognized in epidemiology and public health and point out the importance of institutional structures in relation to health. These institutions are entities acting on the meso level, in contrast to the micro (individual) level and macro (society) levels [17]. In the current review, we focused on family as a significant primary context for school-aged children and adolescents that fundamentally influences the development of health during these life stages. At the same time, the family is influenced by social conditions [18]. Hence, families can be regarded as an entity that acts on the meso level and influences the individual health of a child (micro level) while being shaped by macro level processes (e.g., the general economic situation, social security regulations or labor and employment laws).

Various family characteristics (e.g., parenting practices, family connectedness, parental behavior and family structure) have been shown to directly affect children’s health outcomes [16,19,20,21,22,23]. At the same time, certain family characteristics, like parenting values and practices or family structure, which are linked to children’s health outcomes, are associated with families’ SEP [20,24]. However, less is known about the interplay between families’ SEP, family characteristics and children’s health outcomes. In general, family characteristics can mediate the effects of SEP factors on health outcomes and therefore explain the association. For example, in a hypothetical study the family socioeconomic position is significantly associated with the BMI of the child. In this study, the association can be explained by a third variable, in this case parental unhealthy eating habits. Therefore, the association is regarded as being mediated by parental unhealthy eating habits [25,26]. Family characteristics might also moderate the association between SEP and health outcomes, indicating that the association differs by family characteristics. Using the same example, this would mean that the association between family socioeconomic status and BMI of the child differs by parental unhealthy eating habits. The association would be different depending on the eating habits of the parents [26].

To date, few theoretical frameworks have been developed to describe the mediating or moderating roles of family characteristics in the association between SEP and health in children and adolescents. The family stress model [27] is relatively widespread, positing that a family’s economic hardship predicts greater economic pressure on parents, which in turn leads to greater emotional distress in parents. Parental distress gives rise to family conflicts and parenting that is high in harshness and low in warmth, which can lead to internalizing and externalizing problems in children and adolescents [24,27]. While this is perhaps the most influential theoretical model and has been supported by empirical studies [24,28] over the last several decades, it is restricted to specific effects of economic hardship on children’s mental health outcomes.

The aim of the current systematic scoping review was to comprehensively map evidence regarding the potential mediating or moderating effects of family characteristics on health inequalities in school-aged children and adolescents (6–18 years) in countries with developed economies [29] in Europe and North America. This is, to our knowledge, the first scoping review that was conducted to answer this research question.

## 2. Materials and Methods

This systematic scoping review followed the recommendations of the Preferred Reporting Items for Systematic reviews and Meta-Analyses extension for Scoping Reviews (PRISMA-ScR) [30]. The purpose of a scoping review is to comprehensively map the evidence and assess the scope of the existing literature rather than e.g., estimate a precise point estimate across different studies. Therefore, a scoping review typically includes different study designs, statistical methods and outcomes [30,31]. The study protocol was previously registered in PROSPERO (CRD42020165614) [32]. A cooperating research group simultaneously conducted a similar scoping review of evidence regarding the effects of family characteristics on health inequalities during early childhood (0–6 years) [33].

### 2.1. Eligibility Criteria

To address the objectives of this review, studies were included if they focused on children and adolescents between 6 and 18 years old, living in a private household with at least one parent or step-parent in a country with a developed economy [29] in Europe or North America. We only included studies that analyzed the mediating or moderating effects of family characteristics on SEP-related health inequalities and reported original research results from analyses that were conducted at the individual or family level using individual data. Evidence from quantitative (cross-sectional, cohort, prospective, and case-control studies, as well as baseline data from intervention studies) and qualitative studies was considered eligible for inclusion. Literature reviews were screened for eligible research articles not yet identified by the search. The new articles were included if they matched eligibility criteria. Reviews were excluded. Only peer-reviewed research articles were eligible for inclusion.

The main outcomes of interest were socioeconomic inequalities in subjective health, wellbeing and health-related quality of life, physical health, mental health and health behaviors (e.g., smoking, alcohol consumption, nutrition and physical activity). The measures used to report either socioeconomic inequalities or health outcomes were not restricted due to the anticipated heterogeneity of the included studies, but studies had to either use individual level data or measures on the family level to be included.

Publications were considered eligible for inclusion if published in English or German between 1 January 2000 and 24 June 2021.

### 2.2. Search Strategy

We searched the PubMed, PsycINFO and Scopus electronic databases on 15 January 2000. We updated our search on 24 June 2021.

The search strategy was drafted by one researcher (BW) after initial discussion and agreement within the review team. The search strategies used the following linked concepts: (1) the exposure (SEP); (2) the mediator or moderator (family characteristics); (3) the outcome (health of school-aged children and adolescents); (4) children and adolescents. Within each concept, the terms were combined using the Boolean Operator OR. Additionally, we limited the search to publications between 1 January 2000 and 24 June 2021. The search strategy was further developed in cooperation with a scientific librarian and refined during team discussions. An initial search strategy was developed for PubMed using the Medical Subject Headings (MeSH) thesaurus in addition to relevant free-text terms. The finalized search strategy was then adapted to the other databases. The full search strategy applied to PubMed is shown in Appendix A.

### 2.3. Selection of Sources of Evidence

The identified articles were exported and de-duplicated using Endnote software. The remaining articles were subsequently uploaded to Rayyan, an online tool for managing the study selection process [34]. Two reviewers screened the titles and abstracts of all articles independently (BW and SH). The eligible articles were exported to Endnote again and two reviewers screened all full texts independently (BW and MB). Articles were included if they met the previously defined eligibility criteria. The inter-rater agreement between reviewers was assessed by calculating Cohen’s Kappa after each phase of the selection process. Disagreements were resolved by discussion.

### 2.4. Data Charting Process

A standardized data-charting form was jointly developed by two researchers (BW and MB) in an iterative process. After agreement on the form was achieved, the two reviewers extracted and charted the data independently. The final forms were then compared and combined into the final-data charting form. Inconsistencies between the forms were resolved by discussion. The following items were extracted from each included full-text article: author name, year of publication, country, name of each study that was used as data source, study design, study population, age of the study population (mean/median, standard deviation), number of participants, aim/objective of the study, theoretical framework used, measure(s) of SEP, family characteristics analyzed, children’s and adolescents’ health outcomes, control variables in the analyses, main findings of the analyses, mediating or moderating effect of the family (yes/no), statistical method(s) applied. Studies were assigned to the “mediation” category if they explored whether the association between SEP and health outcomes was explained by a family variable, and to the “moderation” category if they explored variations in the association between SEP and health outcomes by family characteristics.

### 2.5. Evidence Synthesis

Data synthesis was performed in three steps. First, family characteristics and health outcomes were grouped according to inductively derived categories. Two researchers (BW and MB) developed and refined these categories in an iterative process. Subsequently, all included articles were summarized in a table using these categories. Second, main descriptive results were summarized graphically. Finally, these preliminary descriptive results were used to narratively synthesize the evidence [35].

## 3. Results

**Figure 1:**
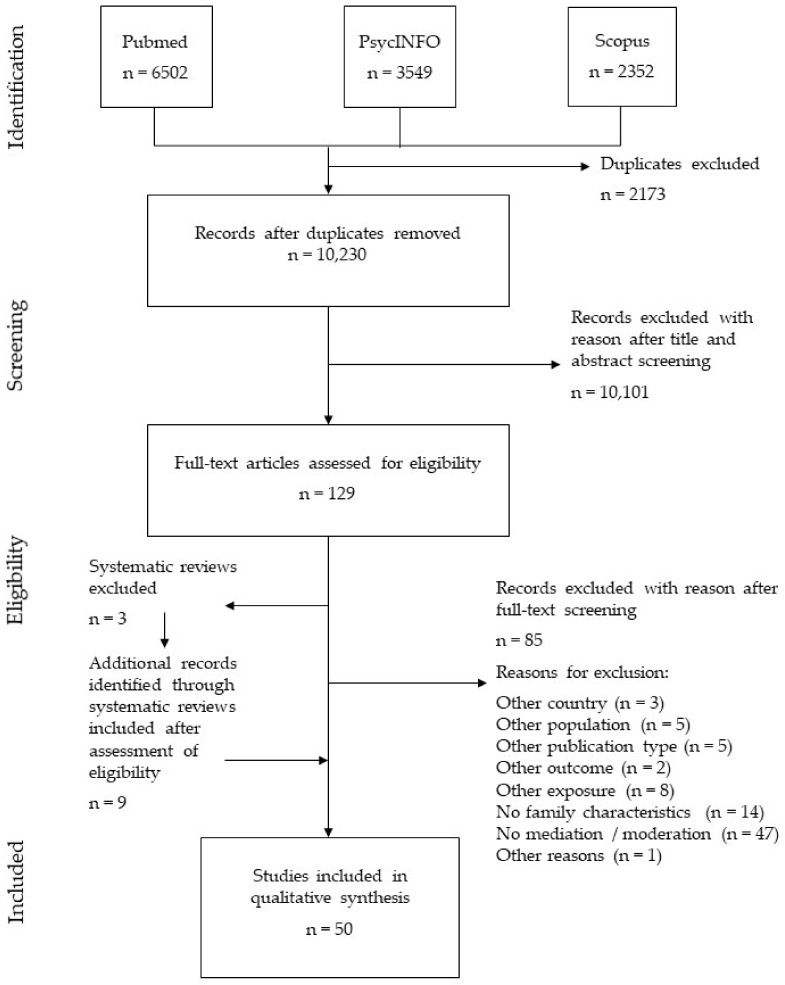
PRISMA flow diagram depicting the study selection process.

Of the 12,403 records identified through our database searches, a total of 50 studies were included in the final synthesis. Figure 1 shows the study selection process following the PRISMA recommendation. Cohen’s Kappa coefficient was 0.95 for the title/abstract screening, and 0.64 for the full-text screening, indicating very good agreement in the first screening phase and good agreement in the second screening phase [36].

The included studies were conducted in the United States (US) (*n* = 27), Europe (*n* = 18), Canada (*n* = 3), or incorporated data from several countries (*n* = 2). The majority of studies were published after 2010 (*n* = 31), while 19 studies were published between 2000 and 2010. Studies used either cross-sectional study designs (*n* = 27) or longitudinal designs (*n* = 23). The overall mean age of the population under investigation in all studies combined was 12.96 years with a median of 13.98. The mean study size of all included studies was 4524 with a median of 1137. The smallest study included 50 participants and the largest study included 52,907 participants. Our search yielded only studies in English language.

The measures used to assess families’ SEP were heterogeneous and included parental income, economic hardship of the family, parental employment status, parental socioeconomic status indices, poverty, parental education, household crowding, financial deprivation, financial stress and family affluence.

Family characteristics were assessed using heterogeneous measures. Based on the included studies, a total of six family categories were derived within the evidence synthesis. Studies were subsequently allocated to the following categories:

- parenting practices (parenting behavior, parenting style, feeding practices, effective parenting, parental involvement, parental support, family environment, parental monitoring, parental acceptance, parental control) [37,38,39,40,41,42,43,44,45,46,47,48,49,50,51,52,53,54,55,56,57,58,59,60,61,62,63,64,65,66],
- parental mental health (mental health, depressive symptoms, anxiety, distress of the parents) [37,39,40,41,45,46,48,49,50,51,57,59,60,64,67,68,69,70,71,72,73],
- parent-child relationship (parent-child interaction, family functioning, perception of parents, family climate, communication, interaction) [40,42,43,53,59,60,63,69,70,71,73,74,75,76,77,78,79,80],
- family structure (marital status, family size, single-parenthood) [39,40,61,66,68,76,81,82],
- parental health and health behavior (smoking, physical activity, TV watching, body mass index (BMI), doctor visiting, meal routines, general health, eating habits) [38,56,65,75,78,82,83,84] and
- family conflict and distress (interparental conflict, family stress, negative life events, family conflicts) [49,50,54,57,80,85,86].

Most frequently, the role of family characteristics on health inequalities was assessed by parenting practices (*n* = 2). Parental mental health (*n* = 22) and parent-child relationship (*n* = 18) were frequently explored as well. Family structure (*n* = 9), parental health and health behavior (*n* = 8) as well as family conflict and distress (*n* = 7) were less frequently analyzed.

School-aged children’s and adolescents’ health outcomes were categorized into the following six categories:

- mental health [39,40,41,43,44,45,46,47,48,49,50,54,55,57,59,60,61,62,63,64,67,68,69,70,71,72,73,79,80,81,85,86],
- substance use [48,52,75,76,78,84],
- subjective health [37,58,66,74,77],
- body weight [38,51,56,65,83],
- physical health [42,54,62,82] and
- physical activity [53].

Most studies explored mental health of children and adolescents as the main health outcome (*n* = 31). Other categories were examined less frequently. Only three studies included several health outcomes simultaneously [48,54,62].

**Figure 2:**
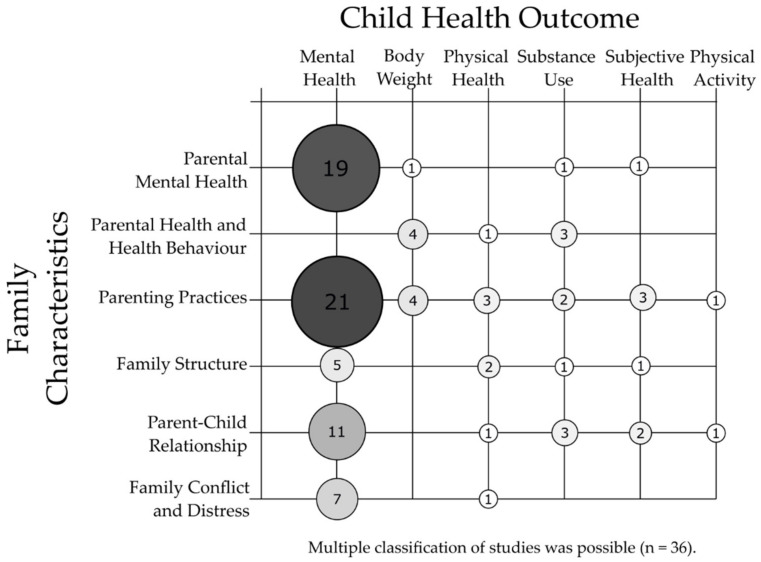
Overview of the combinations of family characteristics on child and adolescent health outcomes in included studies.

Figure 2 shows the combinations of family characteristics and health outcome categories that were analyzed by the included studies. Most studies analyzed parenting practices and mental health of their children when exploring health inequalities. The combination of parental mental health and children’s or adolescents’ mental health was also often analyzed. In addition, family structure, parent-child relationship, as well as family conflict and distress, were often investigated in combination with children’s or adolescents’ mental health. Other combinations of family characteristics and children’s and adolescents’ health were analyzed less frequently.

Approximately half of all studies explicitly used theoretical frameworks for planning the analyses. Of the 50 included studies, 23 studies based their analyses on the family stress model [39,41,44,45,48,49,50,53,54,55,57,59,63,64,66,68,69,70,71,72,73,85,86]. Other socialization theories were used frequently [53,63,64,67,68,76,77]. Focusing on studies using the family stress model, all studies investigated the mental health of children and adolescents as the main health outcome. Some studies explored mental health in combination with physical health [54] or substance use [48]. No theoretical framework was specified by 24 studies.

**Figure 3:**
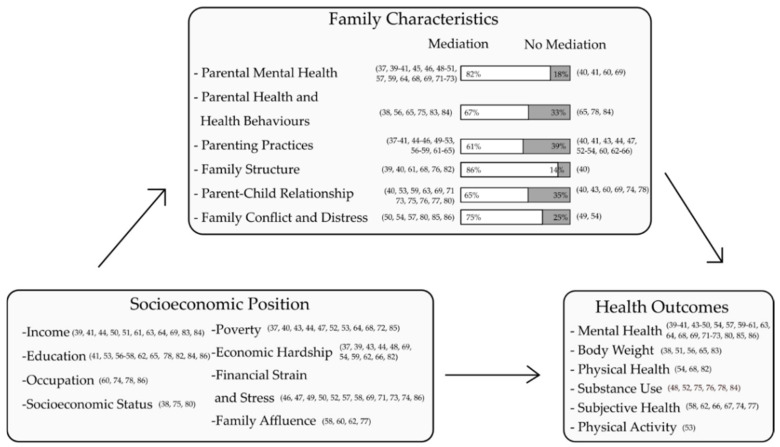
Mediating effects of family characteristics on health inequalities in the included studies.

**Figure 4:**
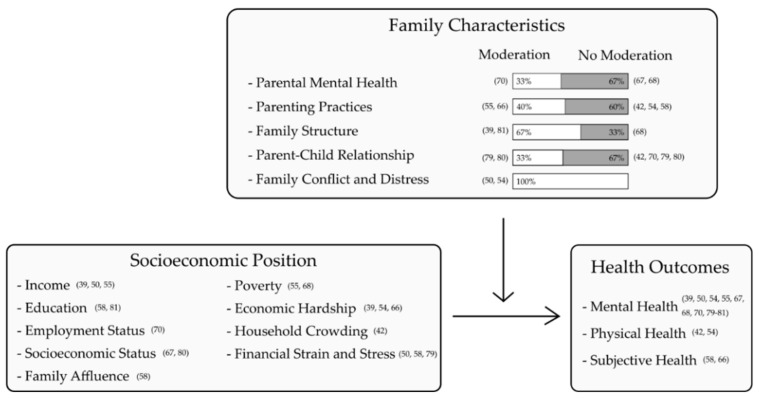
Moderating effects of family characteristics on health inequalities in the included studies.

In the following analyses, we further differentiated whether mediating or moderating effects were analyzed for each family category. A summary of all included studies is shown in Table 1. An overview of the mediating and moderating effects of the family characteristics on health inequalities is shown in Figure 3 and Figure 4.

### 3.1. Mediation Effects

Focusing on the effects of the family context, the majority of the included studies exclusively analyzed mediating effects of different family characteristics (*n* = 37). Seven studies simultaneously investigated mediating and moderating effects (*n* = 44 in total). Across all of these studies, nine different SEP measures were used. The potentially mediating effects of all six previously derived family categories were assessed: parenting practices [37,38,39,40,41,43,44,45,46,47,48,49,50,51,52,53,54,56,57,58,59,60,61,62,63,64,65,66], parental mental health [37,39,40,41,45,46,48,49,50,51,57,59,60,64,68,69,71,72,73], parent-child relationship [40,43,53,59,60,63,69,71,73,74,75,76,77,78,80], parental health and health behavior [38,56,65,75,78,82,83,84], family structure [39,40,61,62,68,76,82], as well as family conflict and distress [49,50,54,57,80,85,86]. In addition, all six different health outcome categories were analyzed. Figure 3 shows an overview of all studies that explored the mediating effects for each family characteristics category and shows whether mediating effects were found or not. Overall, the majority of studies found a mediating effect of different family characteristics on health inequalities in school-aged children and adolescents.

#### 3.1.1. Parenting Practices

Of the 50 included studies, 28 studies analyzed mediating effects of parenting practices on inequalities in different health outcomes. In total, 23 studies found a mediating effect [37,38,39,40,41,44,45,46,48,49,50,51,53,54,56,57,58,59,61,62,63,64,65] and eleven studies found no mediating effect [40,41,43,44,47,52,60,63,64,65,66].

Beiser et al. [40] found that the effect of poverty on internalizing and externalizing problems among offspring was not mediated by parenting practices in an immigrant group, whereas a mediating effect was found in a non-immigrant group [40]. Another study found a mediating effect of maternal warmth and harsh parenting on perceived economic hardship and youth externalizing symptoms, but no mediating effect for paternal parenting [44]. One study showed a mediating effect of parenting stress for the relationship of maternal economic hardship and self-rated health [66]. Another study analyzed the mediating effect of maternal negative communication [62]. The results showed that the association between parental education/financial hardship/economic deprivation and somatic complaints was mediated by maternal negative communication for girls but not for boys. The same effect was found for self-esteem. No mediating effect of maternal negative communication was found for the association between parental education/financial hardship/economic deprivation and depressive symptoms for boys and girls [62]. One study analyzed the mediating effect of parenting practices on the association between parental education and children’s and adolescents’ weight status as well as body composition [65]. They found that shared meals did not mediate the weight status and body composition of five to eleven-year old children. However, a mediating effect was found for 13 to 16-year-old adolescents [65]. Furthermore, media consumption had a mediating effect for 5 to 11-year-old children but not for adolescents. For adolescents and nine to eleven-year-old children, physical activity in a sport club was a mediating factor for this association [65]. Grant et al. [47] found no mediating effect of parenting regarding inequalities in children’s psychological problems [47]. Lee et al. [52] found that the effect of economic strain during childhood on substance use during adolescence was not mediated by parenting [52]. The association between poverty and income and external locus of control, negative self-concept and internalizing behavior of children and adolescents was analyzed in a study by Zhang and Han [64]. They found that all health outcomes were mediated by cognitively stimulating materials. Parent school involvement showed a mediating effect of locus of control and negative self-concept. Parenting style did not mediate the association between poverty or income and child and adolescent mental health [64]. Votruba-Drzal et al. [63] analyzed the mediating effect of cognitive stimulation and corporal punishment. They found that cognitive stimulation did not mediate the association between income and externalizing problems. However, corporal punishment mediated the same association in that less corporal punishment resulted in fewer externalizing symptoms [63]. In another study, negative discipline showed mediating effects depending on whether maternal or paternal education was analyzed [41]. The effects of maternal education and family economy on internalizing and externalizing problems was mediated by negative discipline, whereas this effect was not observed for paternal education [41]. In contrast, a study by Flouri et al. [43] conducted in the UK found no mediation of quality of emotional support and harsh parental discipline for the association between family socio-economic disadvantage and children’s emotional and conduct problems [43].

#### 3.1.2. Parental Mental Health

Of all included studies, 19 investigated the mediating effect of parental mental health. Of those, 13 studies were conducted in the US [37,39,45,46,48,49,50,51,60,64,68,71,72], five were conducted in Europe [41,57,59,69,73] and one was conducted in Canada [40]. The most frequently analyzed outcome in these studies was mental health [39,40,41,45,46,49,50,57,59,60,64,68,69,71,72,73]. Other studies explored mental health and substance use combined [48], body weight [51], or subjective health [37].

Parental mental health most frequently showed a mediating effect in the association between SEP and children’s and adolescents’ health [37,39,40,41,45,46,48,49,50,51,57,59,64,68,69,71,72,73]. However, these effects differed depending on the SEP measures and other variables. Boe et al. [41] reported that the effects of family economic status and maternal education level on externalizing and internalizing problems of adolescents were mediated by paternal and maternal emotional well-being. No mediating effect was found for paternal education and externalizing and internalizing problems [41]. Similar differences between maternal and paternal characteristics were reported by Ponnet [57]. The association between education/financial stress and externalizing behaviors was mediated by maternal and paternal depressive symptoms with some differences between mothers and fathers [57]. In contrast, Layte and McCrory [73] found mediating effects for both paternal and maternal mental health on the association between the families’ objective and subjective economic recession and child psychological adjustment [73]. Zhang and Han [64] found in a longitudinal analysis that parental depressive symptoms have a mediating effect on the association between income or poverty and children’s and adolescents’ external locus of control, negative self-concept and internalizing behavior [64]. Another study reported that the mediating effects varied between regions. Forkel and Silbereisen [69] showed that the effect of SEP on children’s mental health was mediated by parental depressed mood in Western Germany but not in Eastern Germany [69]. However, not all studies consistently reported a mediating effect of maternal depression on health inequalities. Another study not focusing on children’s mental health found that the effect of family income on children’s BMI was mediated by maternal depression [51]. Furthermore, one other study reported that inequalities in subjective health were mediated by parental depression [37]. No mediating effects of optimism and depressive symptomatology were found in a cross-sectional study by Taylor et al. [60] conducted in the US for financial resources and employment on children’s mental health [60].

#### 3.1.3. Parent-Child Relationship

A total of 18 studies explored the mediating effect of the parent-child relationship on health inequalities. Most of these studies reported that the effect of SEP on school-aged children’s and adolescents’ health was mediated by the parent-child relationship [40,53,59,63,69,71,73,75,76,77,80]. Six studies found no mediation of this family category [40,43,60,69,74,78].

The quality of the parent-child relationship was reported by Layte and McCrory [73] to mediate the association between economic recession of the family and children’s psychological adjustment [73]. Another study from the US reported a mediating effect of the parent-child relationship on inequalities in physical activity of adolescents [53]. In this study, parental communication and shared activities completely mediated the effect of family disadvantage on physical activity for females. For males, this mediating effect was also present, but to a lesser extent [53]. The parent-child relationship was also reported to be an important mediator for inequalities in adolescents’ smoking behavior [76]. Furthermore, results from the Health Behaviour in School-aged Children study [77] showed that the relationship with parents, particularly the father, mediated the effect of family affluence on adolescents’ self-rated health. Results of the Millennium Cohort Study showed that the parent-child relationship did not mediate the association between families’ socioeconomic disadvantage and emotional and conduct problems [43]. Another study with data of the Millennium Cohort Study analyzed the mediating effect of parent-child relationship. They found that the association between SEP and externalizing and internalizing symptoms is mediated by parent-child relationship [80]. In addition, parental emotional support has a mediating effect for the association between income and child externalizing behavior problems [63].

Other family characteristics, such as family functioning, were reported to mediate the effect of socioeconomic status on adolescents’ tobacco use [75]. Furthermore, a cross-sectional study from Finland explored economic hardship and mental health of children and adolescents [59]. The results revealed that interaction between parents and children was an important mediator of inequalities in mental health outcomes [59]. Another study suggested that parent-adolescent communication did not mediate the effects of financial resources or employment on depressive symptoms of the child [60].

#### 3.1.4. Family Structure

The role of family structure regarding the association between parental SEP and children’s and adolescents’ health was explored by six studies [39,40,61,68,76,82]. All studies except one [40] found a weaker association when the family structure was included in the modeling [39,61,76,82]. Beiser et al. [40] reported mixed results, observing that single-parent status did not affect the association between poverty and children’s externalizing and internalizing symptoms in an immigrant group while it did affect the association in a non-immigrant group [40]. Studies reporting that health inequalities can, at least in part, be explained by differences in family structure analyzed internalizing and externalizing symptoms [39,40], depressive symptoms [61,68], smoking [76], and respiratory illness [82] of children or adolescents.

#### 3.1.5. Parental Health and Health Behavior

Eight studies investigated the mediating role of parental health and health behavior in the effect of SEP on children’s health or health behavior [38,56,65,75,78,82,83,84]. Five studies consistently found a mediating effect of parents’ health and health behavior on inequalities in the health outcome of the child or adolescent [38,56,65,75,83,84]. Parental health and health behavior were found to mediate inequalities in BMI [38,56], obesity [83], body weight [65], and tobacco use [75,84] of the child or adolescent. Relevant family characteristics in these analyses were parental BMI [38,56,65] or obesity [83], parental tobacco use [75] and parental smoking [65,84]. In contrast to these findings, Ringlever et al. [78] analyzed smoking among Dutch parents and adolescents in a longitudinal study design. They found that the effect of parents’ educational attainment and current occupational status on adolescents’ smoking was not mediated by parental smoking [78]. Similarly, in the UK, Spencer [82] found that the effect of maternal education, material hardship and economic hardship on respiratory illness of the child was not mediated by parental smoking [82].

#### 3.1.6. Family Conflict and Distress

The mediating effect of family conflict and distress was analyzed in seven studies. Of these, five found a mediating effect [50,57,80,85,86] and two found no mediating effect [49,54].

In a cohort study in the US, economic pressure and adolescents’ depressive symptoms were analyzed [49]. The effect of economic pressure was not mediated by parents’ couple conflict [49]. A mediating effect was shown by a study of ninth and eleventh grade African-American adolescents. Family stress explained 50% of the total effect of poverty on adolescents’ depressed mood [85]. In addition, the effects of parental education, parental occupation and economic strain on adolescents’ mental health were mediated by family conflicts [86]. Lee et al. [54] reported that the effects of family economic hardship on youth physical complaints were mediated by marital conflict [54]. Another study by Landers-Potts et al. [50] explored the mediating effect of caregiver relationship conflict and withdrawal on the association between socioeconomic factors and child adjustment and internalizing symptoms. This longitudinal study found a mediating effect of caregiver relationship conflict and withdrawal in an exclusively African-American sample [50]. Another study found that the association between SEP and externalizing and internalizing symptoms is mediated by parent distress [80]. The only non-US study in this category was conducted in Belgium [57]. The analysis revealed that the effects of education and financial stress on externalizing behaviors were mediated by inter-parental conflict [57].

### 3.2. Moderation Effects

The moderating effects of family characteristics were analyzed in a total of 13 studies [39,42,50,54,55,58,66,67,68,70,79,80,81]. An overview of all family characteristics and their moderating effects on health inequalities is shown in Figure 4. For parent-child relationships and parental mental health, the majority of studies found no moderating effects. Regarding family conflict and distress, all studies found a moderating effect.

#### 3.2.1. Parenting Practices

Parenting practice as a moderator variable was analyzed in four studies. Three studies found a moderating effect [42,55], one found no moderating effect [58] and one study found mixed results [54]. Chan et al. [42] found that implicit negative family affect and less implicit warmth exhibited interaction effects with early life SES on resting blood pressure and cholesterol levels. This effect was shown for adolescents with higher early life SES who experienced more implicit negative affect and less implicit warmth, resulting in higher resting blood pressure and cholesterol levels [42]. Furthermore, the results revealed a moderating effect of family support on the association between poverty and internalizing symptoms, as well as a moderating effect of helpfulness of the family on the association between family income/poverty and externalizing symptoms [55]. Adolescents reported fewer internalizing symptoms with high family support and also less externalizing symptoms with more helpfulness of the family [55]. In a US cohort study, the results revealed no moderating effect of supportive parenting and marital conflicts on the association between chronic family economic hardship and physical complaints and anxiety [54]. Another study found no moderating effect of father’s social support on the association of family affluence/perceived financial strain and self-rated health of adolescents [58].

#### 3.2.2. Parental Mental Health

Two studies analyzed the moderating effect of parental mental health on the association between SEP and school-aged children’s and adolescents’ mental health. A study conducted in Portugal reported a moderating effect of parental distress on the association between parental employment status and emotional problems among 15-year-olds. The association between parental unemployment and emotional problems indicated that emotional problems were higher when parental distress was present, and lower in the absence of distress [70]. A study from Denmark reported no moderating effect of lifetime parental psychopathology on the association between family SEP and internalizing and externalizing symptoms of adolescents [67].

#### 3.2.3. Parent-Child Relationship

Four studies analyzed the moderating effect of the parent-child relationship. Two studies found no moderating effect [42,70] and two studies found mixed results [79,80]. A study conducted in Canada by Chan et al. [42] reported that the association between household crowding and metabolic outcomes of adolescents did not differ by parent-child interactions. Furthermore, no moderating effect was found in a study analyzing the role of the parent-child relationship in the association between financial deprivation or parental employment and emotional problems among 15-year-olds in Portugal [70]. Tamura et al. [80] found no moderating effect of parent-child relationship for the association between SEP and children’s and adolescents’ externalizing symptoms. However, they found a moderating effect of parent-child relationship for the association between SEP and internalizing symptoms. In this link, better parent-child relationship in early childhood resulted in lower internalizing symptoms in adolescents [80]. Focusing on relationships with other relatives, a moderating effect of kin social support on the association between financial pressure and adolescent problem behaviors was observed [79]. Thus, when experiencing financial pressure, adolescents with high kin support reported low problem behavior while adolescents with low kin support reported problem behavior. However, no moderating effect of kin support was found for the association between financial pressure and adolescents’ depressive symptoms [79].

#### 3.2.4. Family Structure

Four studies explored the moderating effect of family structure on inequalities for different health outcomes. While three studies reported a moderating effect [39,66,81] one study reported no effect [68]. Three studies explored inequalities in mental health of children and adolescents: internalizing and externalizing symptoms [39], depressive symptoms [68] as well as attention deficit/hyperactivity disorder symptoms and oppositional defiant disorder behaviors [81]. One study analyzed the subjective health of adolescents [66].

A cross-sectional study of seventh- and eighth-grade adolescents found that the association of economic status and perceived economic hardship with internalizing and externalizing symptoms of the adolescents was present in two-parent families, but not in one-parent families [39]. Another longitudinal study conducted in the US reported no differences in the association between poverty and depressive symptoms between one- and two-parent families [68]. A study conducted in Sweden found that family structure moderated the association of education and attention deficit/hyperactivity disorder symptoms and oppositional defiant disorder behaviors. Children of mothers with low education exhibited more symptoms when a new adult moved into the household, compared with children of mothers with low education when no new adult moved into the household. For children of more highly educated mothers, the risk of symptoms was as low as that for children of low-educated mothers when no new adult moved into the household, independently of whether or not another adult moved into the household [81]. Barnhart et al. [66] analyzed the association between maternal economic hardship and adolescents’ self-rated health. This association was moderated by the maternal marital status. For unmarried mothers, an association between economic hardship and self-rated health of their children was found. This association could not be found for married mothers [66].

#### 3.2.5. Family Conflict and Distress

Two studies analyzed family conflict and distress as a moderator for children’s and adolescents’ health inequalities [80]. One study explored the moderating effect of caregiver relationship conflict and withdrawal on the association between family income, negative financial events or economic pressure and child adjustment and internalizing symptoms [50]. This longitudinal study from the US reported a moderating effect in African-American families. Adolescents experienced internalizing symptoms when high levels of caregiver conflict were reported. Low caregiver conflict resulted in lower internalizing symptoms even when economic pressure was experienced [50]. Tamura et al. [80] found no moderating effect of parental distress for the association between SEP and children’s and adolescents’ internalizing and externalizing symptoms [80].

## 4. Discussion

A better understanding of the pathways that can lead to the emergence of health inequalities during childhood and adolescence is urgently needed. Children and adolescents have unequal opportunities to live healthy lives, depending on many factors of their environment and lived experience that are shaped by social determinants. These contextual factors are highly relevant for tackling health inequalities and enabling children and adolescents to live healthy lives with thriving healthy futures. In this scoping review, we closely examined the family as a major contextual factor for health during this life stage. We specifically aimed to systematically map the available evidence on mediating or moderating effects of different family characteristics on the association between families’ SEP and children’s and adolescents’ health in countries with developed economies.

The included studies reported moderating and mediating effects of various family characteristics. Parental mental health, parenting practices and parent-child relationship were most frequently examined, and were found to have important mediating or moderating effects, particularly on mental health inequalities. Family conflict and distress were analyzed less frequently, but were also found to be important mediators or moderators for health inequalities in most studies. In particular, interparental conflicts as well as parent-child conflicts were found to be important mediators in the category of family conflict and distress. Family conflicts, parental mental health and parenting practices were found to be relevant factors identifying pathways in the development of children’s and adolescents’ health inequalities.

As expected, we generally found substantial heterogeneity in SEP measures, family characteristics and health outcomes. A wide range of family characteristics was assessed in the included studies to assess possible effects on children’s and adolescent’s health inequalities. Even studies that used the same terminology captured these characteristics with different scales and questions. This makes the comparison of the studies and their results very difficult and complex. The observed heterogeneity of family characteristics might have occurred because the family entity is highly complex and influences children and adolescents in many ways, as demonstrated in previous studies [16,87]. Therefore, it is reasonable that these studies include several family aspects and members to explore the family in all its complexity. This includes assessing different aspects, such as family structure, parental health behavior and parental mental health.

Parental mental health and parenting practices were the most frequently studied family characteristics. This might be due to the fact that the family stress model was the most frequently used theoretical framework [27]. This theoretical framework focusses on poverty and mental health of children and adolescents, and how parental mental health and parenting practices influence this association. Therefore, it may be unsurprising that the studies in our review mostly explored parental mental health and parenting practices. Several researchers have lately suggested extending the framework of the family stress model on other parental characteristics and economic factors [88,89]. More comprehensive theoretical frameworks that integrate additional measures of SEP, family characteristics and health outcomes might be needed to guide future research and gain a better understanding of the mechanisms and pathways that might influence health inequalities.

To extend current theoretical frameworks, more exploratory research could be useful. The present review identified no qualitative studies. This could be due to the databases we included, but it is also plausible that there is a gap in this research area. To understand the complexity and importance of the characteristics of families in terms of their influence on health, qualitative research might be beneficial to further explore these complexities and guide future quantitative research.

In addition, the current review revealed that school-aged children’s and adolescents’ mental health were by far the most frequently explored outcomes. Few studies analyzed other health outcomes, such as physical health, substance use or subjective health. This situation may have arisen for various reasons. First, the mental health of children and adolescents has mainly been addressed by studies following the family stress model [27]. Second, children and adolescents are rarely affected by non-communicable diseases or chronic conditions. Mental health might be one of the few health outcomes in which health inequalities are detectable at these ages. Third, mental health in general may have received more research attention than other health outcomes over the last two decades [90].

### Strengths and Limitations

To the best of our knowledge, this is the first comprehensive review of the scientific literature on the mediating and moderating effects of family characteristics on health inequalities among school-aged children and adolescents. We used a systematic methodical approach to comprehensively map the available evidence, to guide future research by identifying gaps in current knowledge. However, some possible limitations should be considered when interpreting our results.

In a trade-off between comprehensiveness and manageability, we decided to constrain our search to peer-reviewed publications in three electronic databases with different focus areas: medicine and health sciences, sociology and psychology. Whereas these are the main disciplines related to our research question, we may have missed publications from other research areas like pedagogy, economics or educational sciences. In addition, we focused on peer-reviewed original research and hence excluded grey literature, such as dissertations and reports. This might have led to our results being affected by selection and publication biases, and the inclusion of more studies that found mediating or moderating effects and the neglect of null results. Due to our broad research question that yielded very heterogenous studies that pursued a broad variety of outcomes and measures, a systematic evaluation of the quality of evidence of the included studies was not feasible. Risk of bias assessments and systematic evaluations of the quality of evidence are typically not applicable to scoping reviews [30].We focused on countries with developed economies [29] in Europe and North America, and only included articles published in English or German. Therefore, other countries with developed economies, such as Australia or Japan, were excluded. This approach may limit the generalizability of our results. Overall, more than half of all included studies were conducted in the US, and nine studies exclusively used samples of African-American families. The comparability and generalizability of the results between different countries might be limited as the effects of family characteristics presumably depend on the wider country-specific social context. A study from Brazil indicated e.g., additional family characteristics that were not addressed in the studies we included in our synthesis, such as lack of food at home [91]. To obtain a more comprehensive picture, further research is needed to investigate how different family characteristics have different effects caused by country-specific social regulations, labor market policies, family policies or general social and political characteristics of families. The simultaneous consideration of other contexts, e.g., schools, in addition to the family, might provide a more detailed view [92].

## 5. Conclusions

Current knowledge on the mediating and moderating effects of family characteristics on socioeconomic inequalities in health outcomes in children and adolescents is clustered around a small group of family characteristics: parental mental health, parenting practices and parent-child-relationships. Family conflict was also found to be a relevant factor. These family characteristics might be important targets for interventions to tackle health inequalities that generally arise from structural inequalities, such as income distribution, working conditions and education.

To prevent and reduce health inequalities in childhood and adolescence, a combination of strategies at the micro, meso and macro levels may provide an effective approach. On the one hand, policy interventions and community-based strategies to mitigate poverty and improve employment situations of parents and education of children are important. On the other hand, evidence-based knowledge is needed to elucidate how health inequalities arise in childhood and adolescence and the roles played by the family context in this process. Based on this knowledge, more targeted prevention and health promotion interventions can be developed and specific target groups can be identified (e.g., children of mentally ill parents). Regarding the mental health of young people, for instance, the current review indicated that strengthening parenting skills and promoting the health of parents are promising strategies.

The current review also revealed that further research is needed with regard to family characteristics and other health outcomes, such as health behavior and physical health, to enable more reliable conclusions. A deeper and more comprehensive understanding of the effects of other family characteristics and the inclusion of a wider set of health characteristics of children and adolescents is needed to support future health promotion interventions. In addition, qualitative studies might help to extend current theoretical frameworks to guide future research in this field.

Whereas the importance of the family context for human development during childhood is widely recognized, the additional influences of peers or institutions, such as schools [93] and vocational education and training institutions [94] are becoming more important for children and adolescents as they grow older. Our research should thus be considered in combination with the results of similar analyses of the effects of these institutions to obtain a better understanding of the pathways and mechanisms underlying health inequalities during this life stage.

## Figures and Tables

**Table 1:** Summary table of the study results arranged according to the analyzed categories of family characteristics (L = Longitudinal, C = Cross-sectional, SEP = Socioeconomic Position).

Family Category	Author (Year) [Reference]	Country	Sample Age in Years [Mean, (Range)]	Sample Size	Study Design (L, C)	Measure of SEP	Family Characteristics	Child Health Outcome	Results
**Parental mental health**	Amone-P’Olak (2011) [67]	Netherlands	(11.1–13.6)	2149	L	Family SEP index	Parental lifetime psychopathology	Internalizing and externalizing behavior	No moderation
	Ashiabi (2007) [37]	USA	(6–11)	9645	C	Income poverty, material hardship	Parental depression	Subjective health	Mediation
	Barrera (2002) [39]	USA	12.9 (11–15)	300	C	Objective economic status, perceived economic hardship	Parents depressive symptoms	Internalizing and externalizing symptoms	Mediation
	Beiser (2002) [40]	Canada	(4–11)	13,349	C	Poverty	Parental depression	Externalizing and internalizing problems	Mediation (non-immigrant group)/ no mediation (immigrant group)
	Boe (2014) [41]	Norway	11.8 (11–13)	2043	C	Family economy	Emotional well-being of the children’s caretakers	Internalizing and externalizing problems	Mediation
						Paternal education	Emotional well-being of the children’s caretakers	Internalizing and externalizing problems	No mediation
						Maternal education	Emotional well-being of the children’s caretakers	Internalizing and externalizing problems	No mediation
	Butler (2014) [68]	USA	(12–17; 12–16)	1056	L	Poverty	Mother’s own childhood depression	Depressive symptoms	Mediation
**Parental mental health**	Forkel (2001) [69]	Germany	11.49 (10–13)	304	C	Income, unstable work, income change, can’t make ends meet, material needs, economic adjustments, economic pressure	Parents depressed mood	Depressed mood	Mediation (West Germany)/ no mediation (East Germany)
	Goosby (2007) [45]	USA	12.19 (10–14)	854	C	Poverty	Mothers’ psychological resources	Internalized and externalized behavior	Mediation
	Grant (2000) [46]	USA	12.5 (11–15)	50	C	Economic stress	Maternal depression	Psychological symptoms	Mediation
	Gutman (2005) [71]	USA	13.5 (11–16)	305	C	Income-to-need-ratio, financial strain	Parent psychological distress	Adolescent adjustment	Mediation
	Hardaway (2014) [48]	USA	(10.3; 14.4; 16.5)	300	L	Economic hardship	Maternal psychological distress	Externalizing behavior	Mediation
							Maternal psychological distress	Problem drinking	Mediation
	Henninger (2014) [72]	USA	(10–11)	1067	L	Poverty	Caregiver depression	Externalizing behavior	Mediation
							Parental stress	Externalizing behavior	Mediation
	Kavanaugh (2018) [49]	USA	(13–14; 15–18; 27–30)	451	L	Economic pressure	Maternal depressive symptoms	Depressive symptoms	Mediation
	Landers-Potts (2015) [50]	USA	T1: 10.5 (10–12); T2: 12.5 (12–14)	422	L	Family income, negative financial events index, economic pressure	Parental depressive symptoms	Internalizing symptoms	Mediation
**Parental mental health**	Lane (2013) [51]	USA	(0–11)	1238	L	Income	Maternal depression	BMI	Mediation
	Layte (2018) [73]	Ireland	(9–13)	6272	C	Objective and subjective economic recession	Maternal and paternal depression	Child psychological adjustment	Mediation
	Ponnet (2014) [57]	Belgium	14.27 (11–17)	798	C	Education, financial stress	Depressive symptoms	Externalizing behaviors	Mediation
	Solantaus (2004) [59]	Finland	12.6	527	C	Economic hardship, economic pressure, making ends meet, family expenditures	Maternal and paternal mental health	Mental health	Mediation
	Taylor (2004) [60]	USA	14.74 (12–18)	200	C	Financial resources and employment	Optimism	Depressive symptoms	No mediation
							Depressive symptomatology	Depressive symptoms	No mediation
	Zhang (2021) [64]	USA	Kindergarten, Grade 1, 3, 5, 8	9250	L	Poverty, income volatility	Parenting depressive symptoms	External locus of control, negative self-concept, internalizing behavior	Mediation
**Parental health and health behavior**	Bammann (2017) [38]	Belgium, Germany, Sweden, Estonia, Spain	7.82 (2–11)	5819	C	Socioeconomic status	Parental BMI	BMI	Mediation
**Parental health and health behavior**	Gätjens (2020) [65]	Germany	6.2, 9.9, 14.5	4772	C	Parental education	Maternal and paternal BMI	Weight status and body composition	Mediation
							Smoking habits	Weight status and body composition	Mediation (age 9–16), no mediation (age 5–7)
	Georgiades (2006) [75]	Canada	(12–18)	5401	C	Socioeconomic status	Parental tobacco use	Tobacco use	Mediation
	Jo (2014) [83]	USA	14.22	6913	L	Income	Rules about TV watching, frequency of doctor/dentist visits, and family meal routines	Obesity	Mediation
	Parkes (2016) [56]	Scotland	(3.8–7.5)	2957	L	Maternal education level	Unhealthy eating	BMI	Mediation
	Ringlever (2011) [78]	The Netherlands	15.22	358	L	Parents educational attainment and current occupational status	Smoking status	Adolescents’ smoking behavior	No mediation
							Parents smoking	Adolescents’ smoking behavior	No mediation
	Soteriades (2003) [84]	USA	(12–17)	1308	C	Parental educational attainment and household income	Smoking of parents	Smoking	Mediation
	Spencer (2005) [82]	UK	(0–11)	7926	C	Maternal education, material hardship, family scores on hardship	Maternal smoking	Respiratory illness	No mediation
**Parenting practices**	Ashiabi (2007) [37]	USA	(6–11)	9645	C	Income poverty, material hardship	Positive parenting behavior	Subjective health	Mediation
	Bammann (2017) [38]	Belgium, Germany, Sweden, Estonia, Spain	7.82 (2–11)	5819	C	Socioeconomic status	Parental feeding practices	BMI	Mediation
	Barnhart (2020) [66]	USA	Married mothers: 15.53; unmarried mothers: 15.58	3146	L	Maternal economic hardship	Parenting Stress	Self-rated health	No Mediation
	Barrera (2002) [39]	USA	12.9 (11–15)	300	C	Objective economic status, perceived economic hardship	Parental support	Internalizing and externalizing symptoms	Mediation
	Beiser (2002) [40]	Canada	(4–11)	13,349	C	Poverty	Ineffective parenting	Externalizing and internalizing problems	Mediation (non-immigrant group)/ no mediation (immigrant group)
	Boe (2014) [41]	Norway		2043	C	Maternal education	Negative discipline	Internalizing and externalizing problems	Mediation
						Paternal education	Negative discipline	Internalizing and externalizing problems	No mediation
						Family economy	Negative discipline	Internalizing and externalizing problems	Mediation
**Parenting** **practices**	Chan (2016) [42]	Canada	14.53 (13–16)	259	C	Household crowding	Quality of childhood family environment	Metabolic outcomes	Moderation
	Flouri (2017) [43]	UK	(3–11)	180	L	Family socio-economic disadvantage index	Harsh parental discipline, quality of emotional support	Emotional and conduct problems	No mediation
	Gätjens (2020) [65]	Germany	6.2, 9.9, 14.5	4772	C	Parental education	Shared meals	Weight status and body composition	Mediation (age 13–16), no mediation (age 5–11)
							Media consumption	Weight status and body composition	Mediation (age 5–11), no mediation (age 13–16)
							Physical activity in a sport club of children	Weight status and body composition	Mediation (age 9–16), no mediation (age 5–7)
	Gonzales (2011) [44]	USA	10.43 (9–12)	750	L	Perceived Economic Hardship	Warm and harsh parenting	Internalizing symptoms	No Mediation
								Externalizing symptoms	Mediation
	Goosby (2007) [45]	USA	12.19 (10–14)	854	C	Poverty	Mastery	Internalized and externalized behavior	Mediation
							Parental support	Internalized and externalized behavior	Mediation
	Grant (2000) [46]	USA	12.5 (11–15)	50	C	Economic Stress	Parenting	Psychological symptoms	Mediation
	Grant (2005) [47]	USA	12.89 (11–15)	105	C	Poverty, economic stressors	Neglectful/ distant parenting and inconsistent discipline	Psychological symptoms	No mediation
	Hardaway (2014) [48]	USA	10.3 / 14.4 / 16.5	300	L	Economic hardship	Supportive parenting	Externalizing behavior & problem drinking	Mediation
**Parenting practices**	Kavanaugh (2018) [49]	USA	(13–14; 15–18; 27–30)	451	L	Economic pressure	Mother harsh parenting	Depressive symptoms	Mediation
	Landers-Potts (2015) [50]	USA	T1: 10.5 (10–12); T2: 12.5 (12–14)	422	L	Family income, negative financial events index, economic pressure	Nurturant-involved parenting	Internalizing symptoms	Mediation
	Lane (2013) [51]	USA	(0–11)	1238	L	Income	Parenting behavior	BMI	Mediation
						Income	Parenting style	BMI	Mediation
	Lee (2013) [52]	USA	(7–17)	1285	L	Economic strains	Parenting	Regular smoking	No mediation
							Parenting	Heavy episodic drinking	No mediation
							Parenting	Marijuana use	No mediation
	Lee (2013) [54]	USA	12.7	451	L	Chronic family economic hardship	Parenting support	Anxiety symptoms	Mediation, no moderation
						Chronic family economic hardship	Parenting support	Physical complaints	No mediation, no moderation
						Chronic family economic hardship	Parenting support	Depressive symptoms	Mediation
	Lee (2014) [53]	USA	Female 14.89; male 15.10 (11–20)	9799	L	Family socioeconomic disadvantage	Parental control	Physical activity	Mediation
**Parenting practices**	Li (2007) [55]	USA	11.95 (10–15)	263	C	Family income, poverty level	Family support	Externalizing and Internalizing Symptoms	Moderation
						Family income, poverty level	Helpfulness of family	Externalizing and Internalizing Symptoms	Moderation
	Parkes (2016) [56]	Scotland	(3.8–7.5)	2957	L	Maternal education level	Positive mealtime interaction	BMI	Mediation
							Informal mealtime setting	BMI	Mediation
							Bedroom TV	BMI	Mediation
	Ponnet (2014) [57]	Belgium	14.27 (11–17)	798	C	Education, financial stress	Positive parenting	Externalizing behaviors	Mediation
	Salonna (2012) [58]	Slovakia	16.85	1865	C	Family affluence	Social support of father	Self-rated health of girls and boys	Mediation/ no moderation
						Perceived financial strain	Social support of father	Self-rated health of boys	Mediation/ no moderation
	Solantaus (2004) [59]	Finland	12.6	527	C	Economic hardship, economic pressure, making ends meet, family expenditures	Parenting quality	Mental health	Mediation
	Taylor (2004) [60]	USA	14.74 (12–18)	200	C	Financial resources and employment	Family organization	Depressive symptoms	No mediation
	Tracy (2008) [61]	USA	(11–13)	457	L	Income	Parental support	Depressive symptoms	Mediation
	Votruba-Drzal (2020) [63]	USA	9.09	17,600	L	Income	Corporal punishment	Externalizing behavior problems	Mediation
**Parenting practices**	Walper (2009) [62]	Germany	14.2 (9–19)	358	C	Parental education, financial hardship, economic deprivation	Maternal negative communication	Somatic complaints, self esteem	Mediation (girls) / no mediation (boys)
								Depressive symptoms	No mediation
	Zhang (2021) [64]	USA	Kindergarten, Grade 1, 3, 5, 8	9250	L	Poverty and income volatility	Parenting style	External locus of control, negative self-concept, internalizing behavior	No mediation
							Cognitively stimulating materials	External locus of control, negative self-concept, internalizing behavior	Mediation
							Parent school involvement	External locus of control, negative self-concept	Mediation
								Internalizing behavior	No mediation
**Family structure**	Barnhart (2020) [66]	USA	Married mother: 15.53; unmarried mothers 15.58	3146	L	Maternal economic hardship	Married/ unmarried mothers	Self-rated health	Moderation
	Barrera (2002) [39]	USA	12.9 (11–15)	300	C	Objective economic status, perceived economic hardship	One or two-parent families	Internalizing and externalizing symptoms	Mediation, moderation
	Beiser (2002) [40]	Canada	(4–11)	13,349	C	Poverty	Single-parent status	Externalizing and internalizing problems	Mediation (non-immigrant group)/ no mediation (immigrant group)
**Family structure**	Butler (2014) [68]	USA	(12–17; 12–16)	1056	L	Poverty	Single- and two-parent families	Depressive symptoms	No moderation
	Moor (2015) [76]	35 European and North American countries	15	52,907	C	Family affluence scale	Family structure	Smoking	Mediation
	Rydell (2010) [81]	Sweden	10	1206	C	Education	Family structure	ADHD symptoms and ODD symptoms	Moderation
	Spencer (2005) [82]	UK	(0–11)	7926	C	Maternal education, material hardship, family scores on hardship	Lone parenthood	Respiratory illness	Mediation
	Tracy (2008) [61]	USA	(11–13)	457	L	Income	Marital status	Depressive symptoms	Mediation
						Income	number of adults living in the household	Depressive symptoms	Mediation
	Walper (2009) [62]	Germany	14.2 (9–19)	358	C	Parental education, financial hardship, economic deprivation	Family structure	Adolescent well-being	Mediation
**Parent-Child-Relationship**	Bacikova-Sleskova (2015) [74]	Slovakia	14.3 (11–17)	2799	C	Parental employment status, financial strain	Closeness	Subjective health status	No mediation
							Conflict	Subjective health status	No mediation
							Support	Subjective health status	No mediation
							Communication	Subjective health status	No mediation
							Perception of parents	Subjective health status	No mediation
							Monitoring	Subjective health status	No mediation
	Beiser (2002) [40]	Canada	(4–11)	13,349	C	Poverty	Family dysfunction	Externalizing and internalizing problems	Mediation (non-immigrant group)/ no mediation (immigrant group)
	Chan (2016) [42]	Canada	14.53 (13–16)	259	C	Household crowding	Quality of childhood family environment	Metabolic outcomes	No moderation
	Flouri (2017) [43]	UK	(3–11)	180	L	Family socio-economic disadvantage index	quality of the parent–child relationship	Emotional and conduct problems	No mediation
	Forkel (2001) [69]	Germany	11.49 (10–13)	304	C	Income, unstable work, income change, can’t make ends meet, material needs, economic adjustments, economic pressure	Positive family climate	Depressed mood	Mediation (West Germany), no mediation (East Germany)
**Parent-Child-Relationship**	Tamura (2020) [80]	UK	(9 months to 14 years)	14,452	L	SEP (household income, maternal education and maternal occupation)	Child-parent relationship	Externalizing symptoms	Mediation, no moderation
							Child-parent relationship	Internalizing symptoms	Mediation, moderation
	Frasquilho (2016) [70]	Portugal	15	112	C	Employment Status	Parent-youth-relationship	Emotional problems	Moderation
	Georgiades (2006) [75]	Canada	(12–18)	5401	C	Socioeconomic status	Family functioning	Tobacco use	Mediation
	Gutman (2005) [71]	USA	13.5 (11–16)	305	C	Income-to-need-ratio, financial strain	Parent-adolescent relations	Adolescent adjustment	Mediation
	Layte (2018) [73]	Ireland	(9–13)	6272	C	Objective and subjective economic recession	Parent-child relationship	Child psychological adjustment	Mediation
	Lee (2014) [53]	USA	Female 14.89; male 15.10 (overall 11–20)	9799	L	Family socioeconomic disadvantage	Parental control	Physical activity	Mediation
	Moor (2015) [76]	35 European and North American countries	15	52,907	C	Family affluence scale	Relationship with parents	Smoking	Mediation
**Parent-Child-Relationship**	Moor (2014) [77]	28 European and North American high income countries	(11–15)	29,920	C	Family affluence scale	Relationship to mother and father	Self-rated health	Mediation
	Ringlever (2011) [78]	The Netherlands	15.22	358	L	Parents educational attainment and current occupational status	Frequency of communication, quality of communication	Adolescents’ smoking behavior	No mediation
	Solantaus (2004) [59]	Finland	12.6	527	C	Economic hardship, economic pressure, making ends meet, family expenditures	Marital interaction	Mental health	Mediation
	Taylor (2004) [60]	USA	14.74 (12–18)	200	C	Financial resources and employment	Parent-adolescent communication	Depressive symptoms	No mediation
	Taylor (2014) [79]	USA	14.54 (14–18)	200	C	Financial pressure	Kin social support	Adolescent depressive symptomatology	No moderation
							Kin social support	Adolescent problem behavior	Moderation
	Votruba-Drzal (2020) [63]	USA	9.09	17,600	L	Income	Emotional support	Externalizing symptoms	Mediation
**Family Conflict and Distress**	Hammack (2014) [85]	USA	median 15 (13–18)	1704	C	Poverty	Family stress	Depressed mood	Mediation
	Kavanaugh (2018) [49]	USA	(13–14; 15–18; 27–30)	451	L	Economic pressure	Couple conflict	Depressive symptoms	No mediation
	Landers-Potts (2015) [50]	USA	T1: 10.5 (10–12); T2: 12.5 (12–14)	422	L	Family income, negative financial events index, economic pressure	Caregiver relationship conflict and withdrawal	Internalizing symptoms	Mediation, moderation
	Lee (2013) [54]	USA	12.7	451	L	Chronic family economic hardship	Marital conflict	Anxiety symptoms	No mediation
							Marital conflict	Depressive symptoms	No mediation
							Marital conflict	Physical complaints	No mediation
	Ponnet (2014) [57]	Belgium	14.27 (11–17)	798	C	Education, financial stress	Interparental conflict	Externalizing behaviors	Mediation
	Tamura (2020) [80]	UK	(9 months to 14 years)	14,452	L	SEP (household income, maternal education and maternal occupation)	Parent distress	Externalizing symptoms	Mediation, no moderation
							Parent distress	Internalizing symptoms	Mediation, no moderation
	Wadsworth (2002) [86]	USA	14.7	364	C	Parental education, parental occupation, economic strain	Family conflicts	Coping with stress	Mediation
							Family conflicts	Emotional and behavioral problems	Mediation

## Data Availability

No new data were created or analyzed in this study. Data sharing is not applicable to this article.

## Appendix A. Final Search Strategy for PubMed

Concept (1): Socioeconomic Position

“socioeconomic factors”[MeSH Terms] OR (“socioeconomic”[Title/Abstract] AND “factors”[Title/Abstract]) OR “socioeconomic factors”[Title/Abstract] OR “socio-economic factors” [Title/Abstract] OR (“socio-economic”[Title/Abstract]AND “factor*”[Title/Abstract]) OR (“socioeconomic”[Title/Abstract] AND “factor*”[Title/Abstract]) OR “socioeconomic factor*”[Title/Abstract] OR “socio-economic factor*” [Title/Abstract] OR (“socio-economic”[Title/Abstract]AND “factor*”[Title/Abstract]) OR “socioeconomics”[Title/Abstract]OR “social economic aspect”[Title/Abstract] OR “social economics”[Title/Abstract] OR “social-economic factor”[Title/Abstract] OR “socio-economic aspect”[Title/Abstract] OR “socio-economics” [Title/Abstract] OR “socioeconomic aspect”[Title/Abstract] OR “socioeconomics”[Title/Abstract] OR

“inequalities”[Title/Abstract]) OR “inequities”[Title/Abstract] OR “disparities”[Title/Abstract] OR “inequal*”[Title/Abstract] OR “inequ*”[Title/Abstract] OR “disparit*”[Title/Abstract] OR

“social gradient” [Title/Abstract] OR (“social”[Title/Abstract] AND “gradient”[Title/Abstract]) OR ((“socioeconomic”[Title/Abstract] OR “socio-economic”[Title/Abstract]) AND “gradient”[Title/Abstract]) OR

“social class”[MeSH Terms] OR (“social”[Title/Abstract] AND “class”[Title/Abstract]) OR “social class”[Title/Abstract]) OR (“social”[Title/Abstract] AND “class*”[Title/Abstract]) OR “social class*”[Title/Abstract]) OR “sociocultural class”[Title/Abstract] “socioeconomic class” OR ((“socioeconomic” [Title/Abstract] OR “socio-economic” [Title/Abstract]) AND “class” [Title/Abstract]) OR “socioeconomic status”[Title/Abstract]OR “socio-economic status” [Title/Abstract] OR ((“socio-economic”[Title/Abstract] OR “socioeconomic”[Title/Abstract]) AND “status”[Title/Abstract]) OR “social status” [Title/Abstract] OR “sociocultural class”[Title/Abstract] OR “social condition”[Title/Abstract] OR “social conditions”[Title/Abstract] OR “social economic status”[Title/Abstract] OR “social rank”[Title/Abstract] OR “social standing”[Title/Abstract] OR “social status”[Title/Abstract] OR

“socioeconomic position”[Title/Abstract] OR ((“socioeconomic”[Title/Abstract] OR “socio-economic” [Title/Abstract]) AND “position”[Title/Abstract]) OR “socioeconomic position*”[Title/Abstract] OR ((“socioeconomic”[Title/Abstract] OR “socio-economic” [Title/Abstract]) AND “position*”[Title/Abstract]) OR

“material deprivation”[Title/Abstract] OR (“material”[Title/Abstract] AND “deprivation”[Title/Abstract]) OR “disadvantage”[Title/Abstract] OR “disadvantage*”[Title/Abstract] OR

“poverty”[MeSH Terms] OR “poverty”[Title/Abstract] OR “income”[MeSH Terms] OR “income”[Title/Abstract]) OR “wealth”[Title/Abstract] OR “family affluence”[Title/Abstract] OR ”famil* affluence”[Title/Abstract] OR “famil* affluenc*”[Title/Abstract]OR “economic hardship” [Title/Abstract] OR (“economic”[Title/Abstract] AND “hardship”[Title/Abstract]) OR

“economic status”[MeSH Terms]OR (“economic”[Title/Abstract] AND “status”[Title/Abstract]) OR

“educational status” [MeSH Terms] OR (“educational”[Title/Abstract] AND “status”[Title/Abstract]) OR “educational status”[Title/Abstract] OR “education* status”[Title/Abstract] OR (“education*”[Title/Abstract] AND “status”[Title/Abstract]) OR “educational achievement”[Title/Abstract] OR (“educational”[Title/Abstract] AND “achievement”[Title/Abstract]) OR “educational achievement*”[Title/Abstract] OR (“educational”[Title/Abstract] AND “achievement*”[Title/Abstract]) OR “education” [Title/Abstract] OR

“employment”[MeSH Terms] OR “employment”[Title/Abstract] OR “employment status” [Title/Abstract] OR (“employment”[Title/Abstract] AND “status”[Title/Abstract]) OR “occupations” [MeSH Terms] OR “occupations”[Title/Abstract] OR “occupation*”[Title/Abstract] OR “occupational status”[Title/Abstract] OR(“occupational”[Title/Abstract] AND “status”[Title/Abstract]) OR “occupation* status”[Title/Abstract] OR(“occupation*”[Title/Abstract] AND “status”[Title/Abstract])

AND

Concept (2): Family Characteristics

(“family characteristics”[MeSH Terms] OR “family characteristics”[Title/Abstract] OR (“family”[Title/Abstract] AND “characteristics”[Title/Abstract]) OR (“family”[Title/Abstract] AND “characteristic*”[Title/Abstract]) OR “family context”[Title/Abstract] OR (“family”[Title/Abstract] AND “context”[Title/Abstract]) OR (“family” [Title/Abstract] AND “context*”[Title/Abstract]) OR “family size” [Title/Abstract] OR (“family”[Title/Abstract] AND “size”[Title/Abstract]) OR “family composition” [Title/Abstract] OR (“family”[Title/Abstract] AND “composition”[Title/Abstract]) OR “family structure” [Title/Abstract] OR (“family”[Title/Abstract] AND “structure”[Title/Abstract]) OR (“family”[Title/Abstract]AND “structur*”[Title/Abstract]) OR “family relations”[MeSH Terms] OR “family relations”[Title/Abstract] OR (“family”[Title/Abstract] AND “relations”[Title/Abstract]) OR (“family”[Title/Abstract] AND “relation*”[Title/Abstract]) OR “parenting”[MeSH Terms] OR “parenting”[Title/Abstract] OR (“parents”[Title/Abstract] AND “investments”[Title/Abstract]) OR (“parent”[Title/Abstract] AND “investment*”[Title/Abstract]) OR (“family”[Title/Abstract] AND “stress”[Title/Abstract]) OR (“family”[Title/Abstract] AND “stress*”[Title/Abstract]) OR (“family”[Title/Abstract] AND “cohesion”[Title/Abstract]) OR (“family”[Title/Abstract] AND “support” [Title/Abstract]) OR “child rearing”[Title/Abstract] OR “parent-child relations”[MeSH Terms] OR “parent-child relations”[Title/Abstract] OR “parent-child relation” [Title/Abstract] OR “parent-child relation*” [Title/Abstract] OR “child parent relation”[Title/Abstract] OR “child parent relationship” [Title/Abstract] OR “parent child relationship”[Title/Abstract] OR “parent infant bonding” [Title/Abstract] OR “parent infant relation”[Title/Abstract] OR “parent infant relation*”[Title/Abstract] OR “parental role”[Title/Abstract] OR “parent* role”[Title/Abstract])

AND

Concept (3): Health Outcome

(“subjective health”[Title/Abstract] OR (“subjective”[Title/Abstract] AND “health”[Title/Abstract]) OR “self-rated health” [Title/Abstract] OR “self rated health” [Title/Abstract] OR (“self-rated”[Title/Abstract] AND “health”[Title/Abstract]) OR “srh”[Title/Abstract] OR “self-assessed health”[Title/Abstract] OR “self assessed health”[Title/Abstract] OR “parent-rated health”[Title/Abstract] OR “parent rated health” [Title/Abstract] OR “common mental disorders” [Title/Abstract] OR (“common”[Title/Abstract] AND “mental disorders”[Title/Abstract]) OR “mental disorders”[Title/Abstract] OR “psychiatric disease”[Title/Abstract] OR “psychiatric diseases” [Title/Abstract] OR (“psychiatric” [Title/Abstract] AND “disease*”[Title/Abstract]) OR “depressive disorder”[MeSH Terms] OR (“depressive”[Title/Abstract] AND “disorder”[Title/Abstract]) OR (“depressive”[Title/Abstract] AND “disorders”[Title/Abstract]) OR “depressive disorder”[Title/Abstract] OR “depressive disorder*”[Title/Abstract]OR “depression”[Title/Abstract] OR “depression”[MeSH Terms] OR “depressive symptoms” [Title/Abstract]OR “depressive symptom*”[Title/Abstract] OR “anxiety”[MeSH Terms] OR “anxiety”[Title/Abstract] OR”anxiet*”[Title/Abstract] OR “mental health”[Title/Abstract] OR (“mental”[Title/Abstract] AND “health” [Title/Abstract]) OR “behavioral disorder” [Title/Abstract] OR “behavioral disorder*”[Title/Abstract] OR (“behavioral” [Title/Abstract] AND “disorders” [Title/Abstract]) OR (“behavioral” [Title/Abstract] AND “disorder*” [Title/Abstract]) OR (“behavior*” [Title/Abstract] AND “disorders” [Title/Abstract]) OR “attention deficit”[Title/Abstract] OR “adhs”[Title/Abstract] OR “adhd” [Title/Abstract] OR (“strength”[Title/Abstract] AND “difficulties”[Title/Abstract]) OR “SDQ”[Title/Abstract] OR “obesity”[MeSH Terms] OR “obesity”[Title/Abstract] OR “overnutrition” [Title/Abstract] OR “health related quality of life” [Title/Abstract] OR (“health”[Title/Abstract] AND ”quality”[Title/Abstract] AND “life”[Title/Abstract]) OR “hrqol”[Title/Abstract] OR “exercise”[MeSH Terms] OR “exercise”[Title/Abstract] OR (“physical”[Title/Abstract] AND “activity”[Title/Abstract]) OR “physical activity”[Title/Abstract] OR (“physical” [Title/Abstract] AND “active*”[Title/Abstract]) OR “alcohol drinking”[MeSH Terms] OR (“alcohol”[Title/Abstract] AND “drinking”[Title/Abstract]) OR “alcohol drinking”[Title/Abstract] OR “alcohol consumption” [Title/Abstract] OR (“alcohol”[Title/Abstract] AND “consumption” [Title/Abstract]) OR “smoking”[MeSH Terms] OR “smoking”[Title/Abstract])

AND

Concept (4): Children and Adolescents

“child”[Majr] OR “child”[Title/Abstract] OR “children”[Title/Abstract] OR (“adolescent”[MeSH Terms] OR “adolescent”[Title/Abstract] OR “adolescents”[Title/Abstract]) OR “minors”[MeSH Terms] OR “minors” [Title/Abstract] OR “young people”[Title/Abstract]

## References

[1] CSDH (2008). Closing the Gap in a Generation: Health Equity through Action on the Social Determinants of Health. Final Report of the Commission on Social Determinants of Health.

[2] Pillas D., Marmot M., Naicker K., Goldblatt P., Morrison J., Pikhart H. (2014). Social inequalities in early childhood health and development: A European-wide systematic review. Pediatr. Res..

[3] Nazroo J. (2017). Class and health inequality in later life: Patterns, mechanisms and implications for policy. Int. J. Environ. Res. Public Health.

[4] Lampert T., Hoebel J., Kuntz B., Finger J.D., Hölling H., Lange M., Mauz E., Mensink G., Poethko-Müller C., Schienkiewitz A. (2019). Health inequalities among children and adolescents in Germany. Developments over time and trends from the KiGGS study. J. Health Monit..

[5] Elgar F.J., Pförtner T.-K., Moor I., De Clercq B., Stevens G.W.J.M., Currie C. (2015). Socioeconomic inequalities in adolescent health 2002–2010: A time-series analysis of 34 countries participating in the health behaviour in school-aged children study. Lancet.

[6] Mackenbach J.P., Stirbu I., Roskam A.-J.R., Schaap M.M., Menvielle G., Leinsalu M., Kunst A.E. (2008). socioeconomic inequalities in health in 22 European countries. N. Engl. J. Med..

[7] Currie C., Zanotti C., Morgan A., Currie D., de Looze M., Roberts C., Samdal O., Smith O.R.F., Barnekow V. (2012). Social Determinants of Health and Well-Being among Young People. Health Behaviour in School-Aged Children (HBSC) Study: International Report from the 2009/2010 Survey.

[8] Ben-Shlomo Y., Kuh D. (2002). A life course approach to chronic disease epidemiology: Conceptual models, empirical challenges and interdisciplinary perspectives. Int. J. Epidemiol..

[9] Case A., Fertig A., Paxson C. (2005). The lasting impact of childhood health and circumstance. J. Health Econ..

[10] Siddiqi A., Irwin L.G., Hertzman C., Human Early Learning Partnership, Commission on Social Determinants of Health (2007). Early Child Development: A Powerful Equalizer: Final Report for the World Health Organization’s Commission on the Social Determinants of Health.

[11] Barker D.J. (1995). Fetal origins of coronary heart disease. BMJ Clin. Res. Ed..

[12] Ben-Shlomo Y., Cooper R., Kuh D. (2016). The last two decades of life course epidemiology, and its relevance for research on ageing. Int. J. Epidemiol..

[13] Krieger N. (2011). Epidemiology and the People’s Health: Theory and Context.

[14] Krieger N. (2001). Theories for social epidemiology in the 21st century: An ecosocial perspective. Int. J. Epidemiol..

[15] Blum R.W., Bastos F.I.P.M., Kabiru C.W., Le L.C. (2012). Adolescent health in the 21st century. Lancet.

[16] Viner R.M., Ozer E.M., Denny S., Marmot M., Resnick M., Fatusi A., Currie C. (2012). Adolescence and the social determinants of health. Lancet.

[17] Richter M., Dragano N. (2018). Micro, macro, but what about meso? The institutional context of health inequalities. Int. J. Public Health.

[18] Bronfenbrenner U. (1986). Ecology of the family as a context for human development: Research perspectives. Dev. Psychol..

[19] Raphael D. (1996). Determinants of health of North-American adolescents: Evolving definitions, recent findings, and proposed research agenda. J. Adolesc. Health.

[20] Kramer M.R., Schneider E.B., Kane J.B., Margerison-Zilko C., Jones-Smith J., King K., Davis-Kean P., Grzywacz J.G. (2017). Getting under the skin: Children’s health disparities as embodiment of social class. Popul. Res. Policy Rev..

[21] Mack K.Y., Peck J.H., Leiber M.J. (2015). The effects of family structure and family processes on externalizing and internalizing behaviors of male and female youth: A longitudinal examination. Deviant Behav..

[22] Case A., Paxson C. (2002). Parental behavior and child health. Health Aff..

[23] Rattay P., von der Lippe E., Mauz E., Richter F., Hölling H., Lange C., Lampert T. (2018). Health and health risk behaviour of adolescents—Differences according to family structure. Results of the German KiGGS cohort study. PLoS ONE.

[24] Conger R.D., Conger K.J., Martin M.J. (2010). Socioeconomic status, family processes, and individual development. J. Marriage Fam..

[25] MacKinnon D.P., Fairchild A.J., Fritz M.S. (2007). Mediation analysis. Annu. Rev. Psychol..

[26] Hayes A.F., Rockwood N.J. (2017). Regression-based statistical mediation and moderation analysis in clinical research: Observations, recommendations, and implementation. Behav. Res. Ther..

[27] Conger R.D., Conger K.J., Elder G.H., Lorenz F.O., Simons R.L., Whitbeck L.B. (1992). A family process model of economic hardship and adjustment of early adolescent boys. Child Dev..

[28] Gard A.M., McLoyd V.C., Mitchell C., Hyde L.W. (2020). Evaluation of a longitudinal family stress model in a population-based cohort. Soc. Dev..

[29] United Nations World Economic Situation and Prospects—Statistical Annex. https://www.un.org/development/desa/dpad/wp-content/uploads/sites/45/WESP2020_Annex.pdf.

[30] Tricco A.C., Lillie E., Zarin W., O’Brien K.K., Colquhoun H., Levac D., Moher D., Peters M.D.J., Horsley T., Weeks L. (2018). PRISMA extension for scoping reviews (PRISMA-ScR): Checklist and explanation. Ann. Intern. Med..

[31] Arksey H., O’Malley L. (2005). Scoping studies: Towards a methodological framework. Int. J. Soc. Res. Methodol. Theory Pract..

[32] Wachtler B., Hoffmann S., Rattay P., Sander L. Systematic Review of Qualitative and Quantitative Studies on the Mediating and Moderating Role of Family Characteristics on Health Inequalities in School-Aged Children and Adolescents in Countries with Developed Economies. https://www.crd.york.ac.uk/prospero/display_record.php?ID=CRD42020165614.

[33] Hoffmann S., Wachtler B., Sander L., Blume M., Hilger-Kolb J., Herke M., Matos Fialho P., Pischke C., Novelli A., Lampert T. (2020). Health Inequalities among Infants and Pre-School Children: Protocol for a Scoping Review Examining the Moderating and Mediating Role of Contextual and Compositional Family Characteristics.

[34] Ouzzani M., Hammady H., Fedorowicz Z., Elmagarmid A. (2016). Rayyan—A web and mobile app for systematic reviews. Syst. Rev..

[35] Popay J., Roberts H., Sowden A., Petticrew M., Arai L., Rodgers M., Britten N., Roen K., Duffy S. (2006). Guidance on the Conduct of Narrative Synthesis in Systematic Reviews: A Product from the ESRC Methods Programme.

[36] Grouven U., Bender R., Ziegler A., Lange S. (2007). Der kappa-koeffizient. Dtsch. Med. Wochenschr..

[37] Ashiabi G.S., O’Neal K.K. (2007). Children’s health status: Examining the associations among income poverty, material hardship, and parental factors. PLoS ONE.

[38] Bammann K., Gwozdz W., Pischke C., Eiben G., Fernandez-Alvira J.M., De Henauw S., Lissner L., Moreno L.A., Pitsiladis Y., Reisch L. (2017). The impact of familial, behavioural and psychosocial factors on the SES gradient for childhood overweight in Europe. A longitudinal study. Int. J. Obes..

[39] Barrera M., Prelow H.M., Dumka L.E., Gonzales N.A., Knight G.P., Michaels M.L., Roosa M.W., Tein J.Y. (2002). Pathways from family economic conditions to adolescents’ distress: Supportive parenting, stressors outside the family and deviant peers. J. Community Psychol..

[40] Beiser M., Hou F., Hyman I., Tousignant M. (2002). Poverty, family process, and the mental health of immigrant children in Canada. Am. J. Public Health.

[41] Boe T., Sivertsen B., Heiervang E., Goodman R., Lundervold A.J., Hysing M. (2014). Socioeconomic status and child mental health: The role of parental emotional well-being and parenting practices. J. Abnorm. Child Psychol..

[42] Chan M., Miller G.E., Chen E. (2016). Early life socioeconomic status and metabolic outcomes in adolescents: The role of implicit affect about one’s family. Health Psychol..

[43] Flouri E., Midouhas E., Ruddy A., Moulton V. (2017). The role of socio-economic disadvantage in the development of comorbid emotional and conduct problems in children with ADHD. Eur. Child Adolesc. Psychiatry.

[44] Gonzales N.A., Coxe S., Roosa M.W., White R.M.B., Knight G.P., Zeiders K.H., Saenz D. (2011). Economic hardship, neighborhood context, and parenting: Prospective effects on Mexican-American adolescent’s mental health. Am. J. Community Psychol..

[45] Goosby B.J. (2007). Poverty duration, maternal psychological resources, and adolescent socioemotional outcomes. J. Fam. Issues.

[46] Grant K., Poindexter L., Smith K. (2000). Economic stress and psychological distress among urban African American adolescents: The mediating role of parents. J. Prev. Interv. Community.

[47] Grant K.E., McCormick A., Poindexter L., Simpkins T., Janda C.M., Thomas K.J., Campbell A., Carleton R., Taylor J. (2005). Exposure to violence and parenting as mediators between poverty and psychological symptoms in urban African American adolescents. J. Adolesc..

[48] Hardaway C.R., Cornelius M.D. (2014). Economic hardship and adolescent problem drinking: Family processes as mediating influences. J. Youth Adolesc..

[49] Kavanaugh S.A., Neppl T.K., Melby J.N. (2018). Economic pressure and depressive symptoms: Testing the family stress model from adolescence to adulthood. J. Fam. Psychol..

[50] Landers-Potts M.A., Wickrama K.A.S., Simons L.G., Cutrona C., Gibbons F.X., Simons R.L., Conger R. (2015). An extension and moderational analysis of the family stress model focusing on African American adolescents. Fam. Relat..

[51] Lane S.P., Bluestone C., Burke C.T. (2013). Trajectories of BMI from early childhood through early adolescence: SES and psychosocial predictors. Br. J. Health Psychol..

[52] Lee C.-T., McClernon F.J., Kollins S.H., Prybol K., Fuemmeler B.F. (2013). Childhood economic strains in predicting substance use in emerging adulthood: Mediation effects of youth self-control and parenting practices. J. Pediatr. Psychol..

[53] Lee H. (2014). The role of parenting in linking family socioeconomic disadvantage to physical activity in adolescence and young adulthood. Youth Soc..

[54] Lee T.K., Wickrama K.A.S., Simons L.G. (2013). Chronic family economic hardship, family processes and progression of mental and physical health symptoms in adolescence. J. Youth Adolesc..

[55] Li S.T., Nussbaum K.M., Richards M.H. (2007). Risk and protective factors for urban African-American youth. Am. J. Community Psychol..

[56] Parkes A., Sweeting H., Young R., Wight D. (2016). Does parenting help to explain socioeconomic inequalities in children’s body mass index trajectories? Longitudinal analysis using the growing up in Scotland study. J. Epidemiol. Community Health.

[57] Ponnet K. (2014). Financial stress, parent functioning and adolescent problem behavior: An actor-partner interdependence approach to family stress processes in low-, middle-, and high-income families. J. Youth Adolesc..

[58] Salonna F., Geckova A.M., Zezula I., Sleskova M., Groothoff J.W., Reijneveld S.A., Dijk J.P. (2012). Does social support mediate or moderate socioeconomic differences in self-rated health among adolescents?. Int. J. Public Health.

[59] Solantaus T., Leinonen J., Punamaki R.-L. (2004). Children’s mental health in times of economic recession: Replication and extension of the family economic stress model in Finland. Dev. Psychol..

[60] Taylor R.D., Rodriguez A.U., Seaton E.K., Dominguez A. (2004). Association of financial resources with parenting and adolescent adjustment in African American families. J. Adolesc. Res..

[61] Tracy M., Zimmerman F.J., Galea S., McCauley E., Stoep A.V. (2008). What explains the relation between family poverty and childhood depressive symptoms?. J. Psychiatr. Res..

[62] Walper S. (2009). Links of perceived economic deprivation to adolescents’ well-being six years later. Z. Fam..

[63] Votruba-Drzal E., Miller P., Betancur L., Spielvogel B., Kruzik C., Coley R.L. (2020). Family and community resource and stress processes related to income disparities in school-aged children’s development. J. Educ. Psychol..

[64] Zhang L., Han W.J. (2021). Childhood deprivation experience, family pathways, and socioemotional functioning. J. Fam. Psychol..

[65] Gätjens I., Hasler M., di Giuseppe R., Bosy-Westphal A., Plachta-Danielzik S. (2020). Family and lifestyle factors mediate the relationship between socioeconomic status and fat mass in children and adolescents. Obes. Facts.

[66] Barnhart S., Kilty K.M., Loeffler D. (2021). Family structure as a social determinant of child health. J. Poverty.

[67] Amone-P’Olak K., Burger H., Huisman M., Oldehinkel A.J., Ormel J. (2011). Parental psychopathology and socioeconomic position predict adolescent offspring’s mental health independently and do not interact: The TRAILS study. J. Epidemiol. Community Health.

[68] Butler A.C. (2014). Poverty and adolescent depressive symptoms. Am. J. Orthopsychiatry.

[69] Forkel I., Silbereisen R.K. (2001). Family economic hardship and depressed mood among young adolescents from former East and West Germany. Am. Behav. Sci..

[70] Frasquilho D., de Matos M.G., Marques A., Neville F.G., Gaspar T., Caldas-de-Almeida J.M. (2016). Unemployment, parental distress and youth emotional well-being: The moderation roles of parent-youth relationship and financial deprivation. Child Psychiatry Hum. Dev..

[71] Gutman L.M., McLoyd V.C., Tokoyawa T. (2005). Financial strain, neighborhood stress, parenting behaviors, and adolescent adjustment in urban African American families. J. Res. Adolesc..

[72] Henninger W.R.I.V., Luze G. (2014). Poverty, caregiver depression and stress as predictors of children’s externalizing behaviours in a low-income sample. Child Fam. Soc. Work.

[73] Layte R., McCrory C. (2018). Fiscal crises and personal troubles: The great recession in Ireland and family processes. Soc. Psychiatry Psychiatr. Epidemiol..

[74] Bacikova-Sleskova M., Benka J., Orosova O. (2015). Parental employment status and adolescents’ health: The role of financial situation, parent-adolescent relationship and adolescents’ resilience. Psychol. Health.

[75] Georgiades K., Boyle M.H., Duku E., Racine Y. (2006). Tobacco use among immigrant and nonimmigrant adolescents: Individual and family level influences. J. Adolesc. Health.

[76] Moor I., Rathmann K., Lenzi M., Pfortner T.-K., Nagelhout G.E., de Looze M., Bendtsen P., Willemsen M., Kannas L., Kunst A.E. (2015). Socioeconomic inequalities in adolescent smoking across 35 countries: A multilevel analysis of the role of family, school and peers. Eur. J. Public Health.

[77] Moor I., Rathmann K., Stronks K., Levin K., Spallek J., Richter M. (2014). Psychosocial and behavioural factors in the explanation of socioeconomic inequalities in adolescent health: A multilevel analysis in 28 European and North American countries. J. Epidemiol. Community Health.

[78] Ringlever L., Otten R., de Leeuw R.N.H., Engels R.C.M.E. (2011). Effects of parents’ education and occupation on adolescent smoking and the mediating role of smoking-specific parenting and parent smoking. Eur. Addict. Res..

[79] Taylor R.D., Budescu M., Gebre A., Hodzic I. (2014). Family financial pressure and maternal and adolescent socioemotional adjustment: Moderating effects of kin social support in low income African American families. J. Child Fam. Stud..

[80] Tamura K., Morrison J., Pikhart H. (2020). Children’s behavioural problems and its associations with socioeconomic position and early parenting environment: Findings from the UK Millennium Cohort Study. Epidemiol. Psychiatr. Sci..

[81] Rydell A.-M. (2010). Family factors and children’s disruptive behaviour: An investigation of links between demographic characteristics, negative life events and symptoms of ODD and ADHD. Soc. Psychiatry Psychiatr. Epidemiol..

[82] Spencer N. (2005). Maternal education, lone parenthood, material hardship, maternal smoking, and longstanding respiratory problems in childhood: Testing a hierarchical conceptual framework. J. Epidemiol. Community Health.

[83] Jo Y. (2014). What money can buy: Family income and childhood obesity. Econ. Hum. Biol..

[84] Soteriades E.S., DiFranza J.R. (2003). Parent’s socioeconomic status, adolescents’ disposable income, and adolescents’ smoking status in Massachusetts. Am. J. Public Health.

[85] Hammack P.L., Robinson W.L., Crawford I., Li S.T. (2004). Poverty and depressed mood among urban African-American adolescents: A family stress perspective. J. Child Fam. Stud..

[86] Wadsworth M.E., Compas B.E. (2002). Coping with family conflict and economic strain: The adolescent perspective. J. Res. Adolesc..

[87] Dashiff C., DiMicco W., Myers B., Sheppard K. (2009). Poverty and adolescent mental health. J. Child Adolesc. Psychiatr. Nurs..

[88] Masarik A.S., Conger R.D. (2017). Stress and child development: A review of the Family Stress Model. Curr. Opin. Psychol..

[89] Barnett M.A. (2008). Economic disadvantage in complex family systems: Expansion of family stress models. Clin. Child Fam. Psychol. Rev..

[90] Patalay P., Gage S.H. (2019). Changes in millennial adolescent mental health and health-related behaviours over 10 years: A population cohort comparison study. Int. J. Epidemiol..

[91] Escobar D., Jesus T.F., Noll P., Noll M. (2020). Family and school context: Effects on the mental health of Brazilian students. Int. J. Environ. Res. Public Health.

[92] Morgan K., Melendez-Torres G.J., Bond A., Hawkins J., Hewitt G., Murphy S., Moore G. (2019). Socio-economic inequalities in adolescent summer holiday experiences, and mental wellbeing on return to school: Analysis of the school health research network/health behaviour in school-aged children survey in Wales. Int. J. Environ. Res. Public Health.

[93] Herke M., Moor I., Winter K., Hoffmann S., Spallek J., Hilger-Kolb J., Pischke C., Dragano N., Novelli A., Richter M. (2020). Role of contextual and compositional characteristics of schools for health inequalities in childhood and adolescence: Protocol for a scoping review. BMJ Open.

[94] Matos Fialho P.M., Dragano N., Reuter M., Metzendorf M.-I., Richter B., Hoffmann S., Diehl K., Wachtler B., Sundmacher L., Herke M. (2020). Mapping the evidence regarding school-to-work/university transition and health inequalities among young adults: A scoping review protocol. BMJ Open.

